# Biovalorization of raw agro-industrial waste through a bioprocess development platform for boosting alkaline phosphatase production by *Lysinibacillus* sp. strain APSO

**DOI:** 10.1038/s41598-021-96563-6

**Published:** 2021-09-02

**Authors:** Soad A. Abdelgalil, Nadia A. Soliman, Gaber A. Abo-Zaid, Yasser R. Abdel-Fattah

**Affiliations:** Bioprocess Development Department, Genetic Engineering and Biotechnology Research Institute (GEBRI), City for Scientific Research and Technological Applications, New Borg El-Arab City, Universities and Research Institutes Zone, Alexandria, 21934 Egypt

**Keywords:** Environmental biotechnology, Industrial microbiology, DNA sequencing, Biocatalysis

## Abstract

This study highlighted the exploitation of mathematical models for optimizing the growth conditions that give the highest phosphatase productivity from a newfound *Lysinibacillus* sp. strain APSO isolated from a slime sample. Mathematical models facilitate data interpretation and provide a strategy to solve fermentation problems. Alkaline phosphatase (ALP) throughput was enhanced by 16.5-fold compared to basal medium based on a sequential optimization strategy that depended on two-level Plackett–Burman design and central composite design. The additional improvement for volumetric productivity and specific production yield was followed in a 7 L bench-top bioreactor to evaluate microbial growth kinetics under controlled and uncontrolled pH conditions. The pH-controlled batch cultivation condition neither supported cell growth nor enhanced ALP productivity. In contrast, the uncontrolled pH batch cultivation condition provided the highest ALP output (7119.4 U L^−1^) and specific growth rate (µ = 0.188 h^−1^) at 15 h from incubation time, which was augmented > 20.75-fold compared to the basal medium. To the authors’ knowledge, this study is the second report that deals with how to reduce the production cost of the ALP production process via utilization of agro-industrial waste, such as molasses and food waste (eggshell), as a nutrimental source for the improvement of the newfound *Lysinibacillus* sp. strain APSO ALP throughput.

## Introduction

White biotechnology refers to the usage of modern biotechnology for biochemical, industrial, and bioenergy processing using living cells and/or their enzymes, which is an integral part of environmental sustainability and industrial development and results in safe processes that reduce waste generation and energy use^1^. White biotechnology draws considerable attention from the scientific community for the target-oriented exploration of microbial biocatalysis production for the service of the human healthcare system, industrial applications, ecosystem functioning, and environmental sustainability. Currently, the biotechnological potential of ALP makes it gain enormous attention in white biotechnology^2^. Phosphatases are one of the most crucial enzymes for an organism’s survival and play a vital role in phosphate transportation and metabolism under phosphate-deficient conditions^3^.

Alkaline phosphatase (ALP) [orthophosphoric monoester phosphohydrolase; EC 3.1.3.1] is a ubiquitous, homodimeric, hydrolytic, metallo-dependent, nonspecific, phosphormonoesterase that hydrolyzes a wide variety of phosphoric acid esters (dephosphorylation) to generate free inorganic phosphate at an alkaline pH or move phosphoryl groups into other alcohols and catalyze the phosphorylation reaction in the presence of large amounts of phosphate acceptors^4^. Three strongly spaced metal reticulation positions (M_1_–M_3_) are critical in the enzyme catalytic reaction: two zinc ions (Zn^2+^) occupy sites M_1_ and M_2_ and magnesium ions (Mg^2+^) occupy site M_3_ near the bimetallic center, all of which are essential to the structural stability and catalytic activity of the enzyme^5^. ALP has cosmopolitan distribution among microorganisms, plants, animals, and human tissue. Owing to its intrinsic stability, bacterial ALP tends to be catalytically active for a considerably longer duration than its mammalian counterpart^2^.

Owing to the multiple vital roles of bacterial phosphatases in molecular regulatory activities, cell signaling regulation, and phosphate homeostasis, bacterial phosphatases have gained attention in recent decades, as they appear to have great potential in the fields of molecular biology and immunodetection and are being exploited in environmental and agricultural biotechnology and animal feeding^4^.

To meet the current and forecasted market demand, bacterial ALP production must be increased manifoldly through innovative and efficient production strategies. An increase in productivity reduces the overall cost of the product and the production cost. The key issues of industrial fermentation, process optimization, and scale-up retain optimal and homogeneous reaction conditions that reduce the explanation to microbial stress and maximize metabolic precision to boost product yield and ensure good product quality. With the advent of modern mathematical and statistical techniques, medium optimization has become more vibrant, effective, efficient, economical, and robust in giving the results^6^.

Fermentation modeling and chemometric analysis can describe chemical and physical factors in near-real-time data obtained through online and offline measurements^7^. To limit the number of significant experiments done by employing large-batch and fed-batch bioreactor cultures, the optimal bioprocess development strategy is identified and optimized initially via throughput screening on a small scale (shake-flasks)^8^. For a strain with uncertain comfortability to establish a microbial production process, it is important to identify the physiological features that describe the link between microbial development and ultimate output in a dramatic way. A high initial microbial concentration and a low specific growth rate are often conducive to the optimized development of product^9^. In the employment of enzymes in industrial processes, the related costs are of major significance.

**Figure 1:**
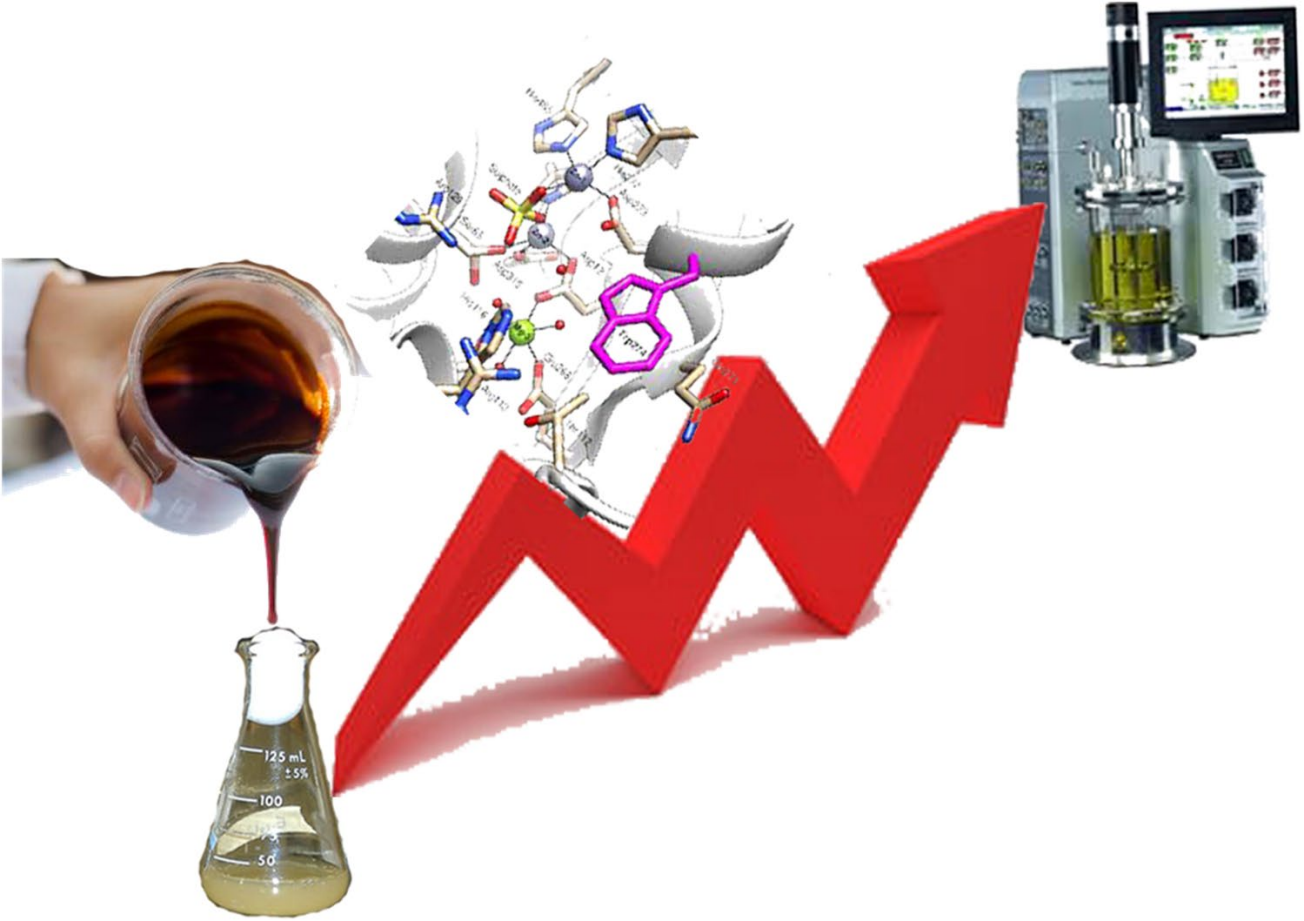
Schematic illustration of the exploitation of green chemistry sustainable strategies for the ultimate benefit of biowaste sugarcane molasses for the scale-up production of bacterial ALP on bench top-scale production.

The large-scale production of enzymes is a capital-intensive process that indirectly affects the cost of the finished product in various manufacturing processes. The scale-up strategy is a complex biochemical process for novel microbial production purposes based on low production cost, low environmental pollution, and high innovation. There have been significant attempts to discover new enzymes from different species to boost current enzymes, refine internal processes, or obtain marketable intellectual property^10^. Scale-up of bacterial ALP production has no received attention to date. According to those mentioned above, this study is an innovative step toward the scale-up of newfound *Lysinibacillus* sp. strain APSO ALP production from shake-flask to bench-top bioreactor scale using biowaste sugarcane molasses, as illustrated in Fig. 1*.*

## Results and discussion

### Isolation and identification of ALP-producing bacteria

**Figure 2:**
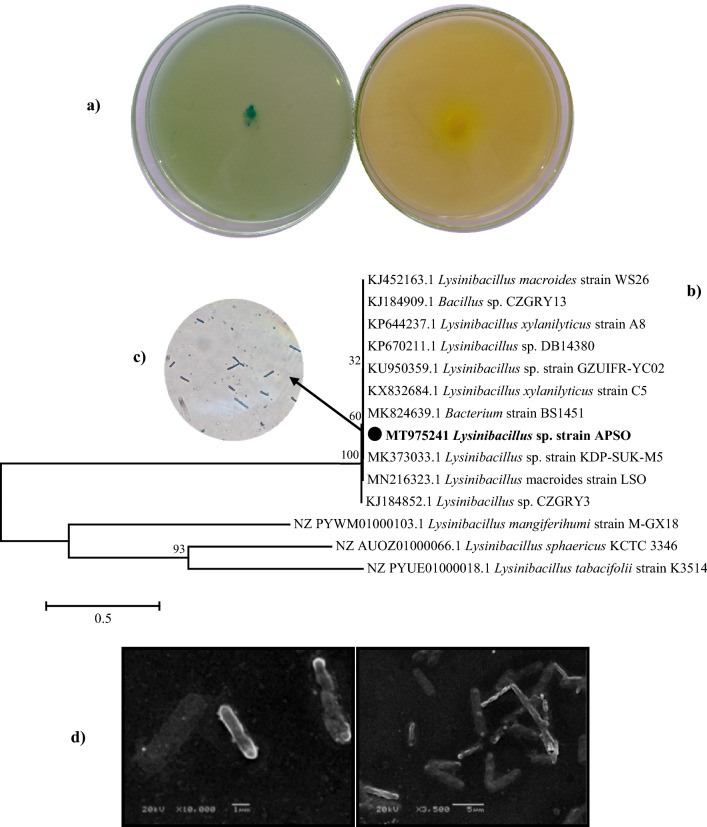
Isolation, screening, and identification of ALP-producing bacteria. **(a)** Qualitative screening of ALP-producing bacteria. **(b)** Phylogenetic tree based on 16S rDNA gene sequence analysis showing the relationship of *Lysinibacillus* sp. strain APSO with reference strains (NCBI GenBank) constructed using the neighbor-joining method with the aid of MEGA 7.0 program. Sequence divergence is indicated by the scale bar. **(c)** Gram stain of *Lysinibacillus* sp. strain APSO (magnification, oil lens, × 100). **(d)** SEM micrograph of *Lysinibacillus* sp. strain APSO showing cell morphology at a magnification of × 3500 and × 1000 with 20 kV.

In the preliminary exploration, the growth of the bacterial microflora of a slime sample from Alexandria paper and pulp factory was enriched in a modified Pikovskaya (PVK) broth^11^ medium supplemented with eggshell powder as a phosphate source. The pure isolates were screened to produce extracellular ALP using chromogenic agar medium, such as methyl green (MG)-phenolphthalein diphosphate (PDP) agar and rich medium supplemented with ρ-nitrophenyl phosphate (ρNPP). The MG-PDP method has a unique advantage over other procedures, through which the contamination opportunity is avoided and the distinction between excreted and cellular phosphatase was more obvious. These media had previously been used to pick out the most potent ALP-producing bacteria, as reported by Patel Falguni et al.^12^. Approximately 30 isolates showed notable ALP activity on MG-PDP agar. Based on the colony color intensity, 4 of 30 isolates exhibited a high capability of ALP productivity. Among these, the colony of the APSO isolate manifested the highest obviousness of green and yellow color intensity, as shown in Fig. 2a, and showed a maximal ALP activity of 145 U L^−1^ min^−1^ compared to the other three isolates that showed enzyme activities of 76.99, 35.93, and 14.6 U L^−1^ min^−1^ according to the quantitative screening program. For this reason, the APSO isolate was picked out for a further detailed investigation. The representative isolate that displayed the greatest activity was identified using molecular identification. Sanger sequencing of the dideoxynucleotide of the amplified *16S rRNA* gene (S.1.)﻿ has resulted in a 1211 bp nucleotide sequence. Analysis based on genome-derived *16S rRNA* gene sequences in this study showed that the APSO isolate has a homology of > 99.92% relative to the genus *Lysinibacillus*. The *16S rRNA* gene sequences have been submitted to the national center for biotechnology information (NCBI) GenBank with accession number MT975241. The *16S rRNA* gene sequences of the reference taxa were retrieved from the NCBI GenBank and aligned using MEGA 7.0 for dendrogram construction. The resultant tree topologies were evaluated by bootstrap analysis based on 500 replicates. The results of this analysis (Fig. 2b) indicated that APSO formed a coherent branch within the genus *Lysinibacillus*, with a bootstrap value of 100%. It also formed a robust cluster with strains of the genus *Arenimonas* and shared the highest sequence similarity with *Lysinibacillus* sp. strain KDP-SUK-M5 (99.92%) and a query cover of 100%, so it could be identified as *Lysinibacillus* sp. strain APSO. *Lysinibacillus* sp. strain APSO was characterized by a rod shape (0.72 wide × 3.45 μm long), Gram-positive staining, and spherical endospore, as shown in Fig. 2c,d. Several earlier studies reported that the *Bacillus* genus is one of the most authoritative ALP producers^4,13^.

### Effects of physical parameters on ALP productivity

One of the factors that control ALP production processes is the physical parameter. Temperature is one of the most critical environmental parameters for controlling the physiological and biological activities of microorganisms; hence, it influences all bioprocess operations^14^. To evaluate the optimal temperature for enhancing ALP productivity, the bacterial culture was incubated at different temperatures (37 °C, 40 °C, and 45 °C) using a modified PVK broth medium^11^ with eggshell powder. The results elucidated that ALP productivity gradually increased from 37 °C (29 U L^−1^) passing by 40 °C (41.5 U L^−1^) until it reached the optimal activity peak at 45 °C with 209.61 U L^−1^. This finding was congruent with earlier observations of Behera et al.^13,15^, who recorded that the maximal phosphatase production by *Serratia* and *Alcaligenes faecalis* was accomplished at 45 °C. Recently, Abdelgalil et al.^16^ reported that the peak of ALP production from *Bacillus paralicheniformis* strainAPSO was achieved at 45 °C.

ALP production depends greatly on the initial pH of the culture medium, as it can influence many enzymatic processes and nutrient transport across the cytoplasmic membrane. This study revealed that the optimal ALP production was recorded at pH 6.0 and 7.0 (149.4 and 231.6 U L^−1^), and the gradual decline in enzyme productivity (147.8, 106.3, and 63.2 U L^−1^) was obtained at pH 8.0, 9.0, and 10.0, respectively. The results were in accordance with Jena et al.^17^ and Dhaked et al.^18^, who found that neutral pH (7.0) is the most suited for high ALP production by *Bacillus* sp. In contrast, Abdelgalil et al.^16^ revealed that *B. paralicheniformis* strain APSO exhibited maximal ALP production at pH 9.0, incompatible with this finding.

To optimize the inoculum size for improving ALP productivity, bacterial cells were grown on a series of activated precultured inoculum sizes (1%, 2%, 5%, and 10%). The results elucidated that a gradual decline in enzyme production was observed as the inoculum size increased. Maximum ALP productivity was obtained using 1% activated precultured inoculum size (343.4 U L^−1^), whereas a gradual decrease in productivity was noted as the inoculum size increased by twofold (166.2 U L^−1^), 2.5-fold (137.1 U L^−1^), and 3.5-fold (94.1 U L^−1^) at 2%, 5%, and 10% inoculum sizes. This finding matched Qureshi et al.^19^, who utilized 1% inoculum size for ALP production by *Escherichia coli* EFRL 13 in submerged fermentation. In contrast, the optimal inoculum size for the highest ALP throughout was recorded at 5% of the preactivated culture of *B. paralicheniformis* strain APSO^16^.

### Statistical optimization of ALP production by *Lysinibacillus* sp. strain APSO

Medium optimization has reached new horizons with the development of statistical technologies, as these strategies increase the productivity of the process, minimize production time and labor expense, and contribute to improving the overall economy of the process. To optimize the ALP production process, the following experimental design strategies are highly recommended: First, Plackett–Burman design (PBD) was employed to evaluate the relative significance of cultivation variables of the respective ALP production system (Screening). Table 1 shows the randomized PBD matrix selected to screen the most significant variables for ALP production along with the corresponding experimental and predicted response (ALP productivity). Table 1 shows a noticeably broad variation in ALP productivity throughout different trials ranging from 14.8 to 959.4 U L^−1^, reflecting the significance of medium optimization for improving ALP productivity. The highest ALP activity was achieved at trial 17 followed by trials 16 and 7 that contained molasses and MgCl_2_·6H_2_O at the highest level. This inferred that molasses and MgCl_2_·6H_2_O are the most significant variables for ALP stimulation by *Lysinibacillus* sp. strain APSO. In contrast, trial 5 that contained the lowest level of molasses and MgCl_2_·6H_2_O showed unremarkable activity (14.8 U L^−1^), demonstrating the significance of molasses and MgCl_2_·6H_2_O on the production process. Analysis of variance (ANOVA) results provided the weight of each independent variable for the investigated response, as shown in Table 2.

**Table 1:** Randomized PBD for evaluating factors influencing ALP production by *Lysinibacillus* sp. strain APSO.

Trials	Variables									ALP productivity (U L^−1^ min^−1^)		
	X_1_	X_2_	X_3_	X_4_	X_5_	X_6_	X_7_	X_8_	X_9_	Actual value	Predicted value	Residual
1	− 1	− 1	− 1	− 1	1	1	− 1	− 1	1	30.228	30.26852	− 0.04052
2	1	− 1	1	− 1	− 1	− 1	− 1	1	1	221.5278	297.0267	− 75.4989
3	− 1	1	− 1	− 1	1	1	1	1	− 1	90.68889	172.3118	− 81.6229
4	1	1	1	− 1	1	− 1	1	− 1	− 1	562.2222	580.8505	− 18.6283
5	− 1	− 1	1	1	− 1	− 1	1	− 1	− 1	14.86222	76.08983	− 61.2276
6	1	− 1	− 1	1	− 1	− 1	1	1	1	250.5556	281.0313	− 30.4757
7	1	1	− 1	1	− 1	1	− 1	− 1	− 1	928.8889	885.1152	43.77368
8	1	1	1	1	1	1	1	1	1	244.4444	326.3203	− 81.8759
9	0	0	0	0	0	0	0	0	0	211.6889	276.7231	− 65.0342
10	− 1	− 1	− 1	1	1	− 1	− 1	1	− 1	25.09613	− 14.3436	39.4397
11	1	− 1	− 1	1	1	1	1	− 1	1	331.5278	432.7664	− 101.239
12	1	− 1	1	− 1	1	− 1	− 1	− 1	− 1	531.6667	553.7209	− 22.0543
13	− 1	1	1	1	1	− 1	1	− 1	1	38.92778	− 169.648	208.5757
14	− 1	− 1	1	1	1	1	− 1	1	− 1	63.55556	44.93015	18.6254
15	− 1	− 1	1	− 1	− 1	1	1	1	1	48.70556	− 112.977	161.6824
16	1	1	1	1	− 1	1	− 1	1	− 1	777.6389	700.7556	76.88334
17	1	− 1	− 1	− 1	− 1	1	1	− 1	− 1	959.4444	789.1495	170.295
18	− 1	1	− 1	1	− 1	− 1	− 1	− 1	1	27.5814	40.55455	− 12.9731
19	− 1	1	1	− 1	− 1	1	− 1	− 1	1	25.58502	132.56	− 106.975
20	1	1	− 1	− 1	1	− 1	− 1	1	1	488.8889	350.5623	138.3266
21	0	0	0	0	0	0	0	0	0	207.7778	276.7231	− 68.9453
22	− 1	1	− 1	− 1	− 1	− 1	1	1	− 1	71.43889	137.416	− 65.9772
23	0	0	0	0	0	0	0	0	0	211.6889	276.7231	− 65.0342

Variable	Code	Coded and actual levels										
		− 1	0	1								
Molasses	X_1_	1	4.5	10								
NaNO_3_	X_2_	0.1	0.3	0.5								
(NH_4_)_3_SO_4_	X_3_	0.1	0.3	0.5								
Eggshell	X_4_	0.2	1.1	2								
NaCl	X_5_	0.1	0.3	0.5								
MgCl_2_·6H_2_O	X_6_	0.02	0.11	0.2								
CuSO_4_·5H_2_O	X_7_	0.0005	0.0015	0.0025								
CoCl_2_·6H_2_O	X_8_	0.0005	0.0015	0.0025								
ZnSO_4_·H_2_O	X_9_	0.0005	0.0015	0.0025

**Table 2:** Statistical analysis of PBD showing coefficient values, *t*-values, and *ρ*-values for each variable affecting ALP production.

Variables	Coefficient	Main effect	SE	*t*-Stat	*P*-value	Confidence level (%)
Intercept	276.7230		25.30831	10.93407	6.32436E^−08^	99.99999
X_1_	243.0068	486.0136	27.14014	8.953775	6.38265E^−07^	99.99993
X_2_	38.95678	77.91356	27.14014	1.435393	0.1747964	82.52035
X_3_	− 33.76013	− 67.5202	27.14014	− 1.24391	0.2354926	76.45073
X_4_	− 16.36588	− 32.7317	27.14014	− 0.60301	0.5568738	44.31261
X_5_	− 45.94911	− 91.8982	27.14014	− 1.69303	0.1142609	88.57390
X_6_	63.39699	126.7939	27.14014	2.33591	0.0361627	96.38372
X_7_	− 25.39197	− 50.78394	27.14014	− 0.93559	0.3665408	63.34591
X_8_	− 58.41969	− 116.8393	27.14014	− 2.15251	0.0507224	94.92775
X_9_	− 115.8765	− 231.7530	27.14014	− 4.26956	0.0009134	99.90865

ANOVA	df	SS	MS	*F*	Significance *F*	
Regression	9	1,711,860	190,206	12.91134	0.0000399	
Residual	13	191,512	14,731			
Total	22	1,903,373				
*R*^*2*^	0.90					
Adjusted *R*^*2*^ Square	0.8297					

**Figure 3:**
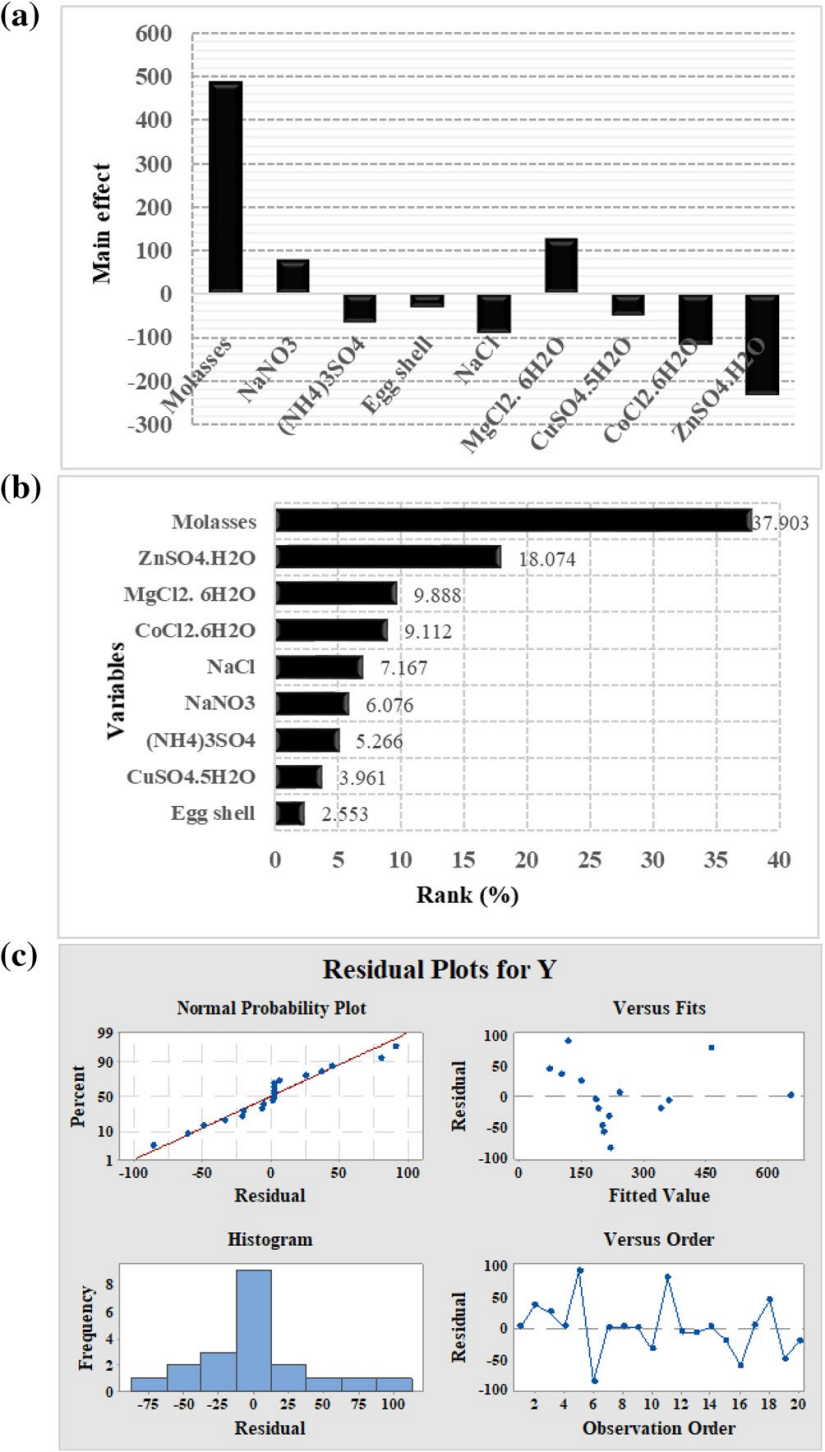
PBD results: **(a)** main effect of culture variables, **(b)** Pareto chart illustrating the order and significance of the variables affecting ALP production by *Lysinibacillus* sp. strain APSO in a ranking percentage from 2.553 to 37.903, and **(c)** normal probability plot of the residuals for ALP production determined by the first-order polynomial equation.

The probability (*p*) value was utilized to conclude the main nutrimental factors at a confidence level of > 95%, as summarized graphically in Fig. 3. The lower probability value (*p* < 0.05) and the larger *t*-value indicated that the process variable significantly affects ALP yield, and *p*-values > 0.05 indicate that the model terms are nonsignificant. Based on ANOVA, the most significant factors that positively influence ALP production were molasses (X_1_) with a contribution ratio of 99.99 and *p* = 0.00000063, followed by MgCl_2_·6H_2_O (X_6_) with a contribution ratio of 96.383 and *p* = 0.036. The third significant variable in the production process was NaNO_3_ (X_2_) with a contribution ratio of 82.5 and *p* = 0.174 (Table 2). The insignificant factors with a low contribution ratio and *p* > 0.05, which negatively influenced the production process, including eggshell powder, CuSO_4_·5H_2_O, (NH_4_)_3_SO_4_, NaCl, CoCl_2_·6H_2_O, and ZnSO_4_·H_2_O, were neglected in a further study. A standardized Pareto chart (Fig. 3b) was employed to determine the influence of the most important parameters. It was constructed by bars with a length proportional to the absolute value of the estimated effects divided by the standard error. The bars are presented in order of the effect size, with the largest effects on top.

In this study, ANOVA of PBD demonstrated that the model was highly significant, as evident from a Fisher’s *F*-test value of 12.91 with a very low probability value (*p* = 0.0000399; Table 2). This implied that there was a statistically significant relationship between the variables at the 99.999999% confidence level. Statistical *R*^2^ showed that the fitted model explained 90% of ALP activity variation. Furthermore, the highest value of the adjusted *R*^2^ statistic (82%; Table 2) indicated that the model was highly significant, and the correlation between experimental variables and ALP productivity was more precise and reliable. The first-order polynomial model describing the correlation between the nine factors and ALP productivity could be expressed as follows:

$${\text{Y}}_{{{\text{activity}}}} = { 276}.{72} + {\text{243X}}_{{1}} + {38}.{\text{95X}}_{{2}} - {33}.{\text{76X}}_{{3}} - {16}.{\text{36X}}_{{4}} - {45}.{\text{94X}}_{{5}} + { 63}.{\text{39X}}_{{6}} - {25}.{\text{39X}}_{{7}} - {58}.{\text{41X}}_{{8}} - {115}.{\text{87X}}_{{9}}$$

The important graphical method to detect and explain whether the obtained data set is normal or deviates from normality is the normal probability plot^20^. In this study, residuals were plotted against the expected normal values of the model, as illustrated in Fig. 3c. The normal probability chart clearly showed that residuals were distributed normally; the points were distributed nearly to the diagonal line. This indicated that the expected ALP productivity was well fitted with experimental results. To assess the accuracy of PBD, a confirmation experiment was performed. The culture conditions predicted to be optimum for maximal ALP production from *Lysinibacillus* sp. APSO were pH 6.45, temperature 45 °C, inoculum size 1% v v^−1^, agitation speed 200 rpm, incubation time 48 h, and medium of the following composition: molasses, 10 g L^−1^; NaNO_3_, 0.5 g L^−1^; and MgCl_2_·6H_2_O, 0.2 g L^−1^. The maximal ALP activity obtained under these optimal conditions was 3208.333 U L^−1^, higher than ALP activity obtained before application of PBD (343 U L^−1^) by ~ 9.3-fold.

There are very few reports on the optimization of bacterial ALP production using statistical and experimental PBD. Pandey et al.^21^ reported the optimization of a physical parameter only for enhancing ALP production by *B. licheniformis*. Glucose, yeast extract, and agitation were reported to enhance maximal ALP production from *Geobacillus thermodenitrificans* I2 isolate, whereas NaCl, ZnSO_4,_ and CuSO_4_ contributed negatively; this finding matched that in this study^22^. The other published articles depended on the one-factor-at-a-time (OFAT) method to optimize bacterial ALP production.

The most published articles used a synthetic medium and applied the OFAT method for bacterial ALP production. In addition, the use of an agro-industrial waste-based medium for ALP production has been poorly investigated. To the authors’ knowledge, Abdelgalil et al.^16^ were the first investigators to utilize an agro-industrial waste to boost ALP production from *B. paralicheniformis* strain APSO. They demonstrated that the most significant variables that directly enhanced ALP production by PBD are molasses, (NH_4_)_2_SO_4_, and KCl; this finding was consistent with this study. Adopting this trend, this study directed to the ultimate beneficial utilization of agro-industrial and food waste and low-cost nutrimental variables for enhancing bacterial ALP output on laboratory-scale production to reduce the cost of the production process through complicated statistical optimization strategies.

Compared to the results obtained by other investigators, Qureshi et al.^19^ reported that 2% sodium nitrate had the strongest influence on ALP from *E. coli* EFRL 13 among other variables used (tryptone, ammonium chloride, and potassium nitrate). Furthermore, molasses (15 g L^−1^) and sodium nitrate were utilized for improving ALP production from rhizospheric bacterial isolates^23^. In contrast to this study, Parhamfar et al.^11^ documented that maximal ALP production from *Bacillus* spp. was achieved by glucose (10 g L^−1^) and ammonium sulfate (5 g L^−1^) as the carbon and nitrogen source. Additionally, Jatoth et al.^4^ found that starch (5 g L^−1^) and egg albumin (5 g L^−1^) were suited and acted as enhancers of ALP production from *B. subtilis*.

### Response surface methodology (RSM)

Based on the fit of empirical models to the experimental data in relation to the experimental design, collections of mathematical and statistical approaches expressed as RSM are utilized. To maintain high-efficiency and profitable bioprocesses, microbial industry sponsors are recommended to utilize RSM approaches during production^24^. To determine the optimal response region of ALP productivity, the most significant independent variables that positively affect ALP production as identified by PBD design results (molasses, NaNO_3_, and MgCl_2_·6H_2_O with contribution percentages of 99.99%, 82.52%, and 96.38%, respectively) were explored more via the application of central composite design (CCD) with uniform precision by five levels for each parameter. Consequently, the intercorrelations among independent variables and their optimal levels were evaluated via regression analysis of this approach.

Table 3 illustrates the array of the design matrix for a total number of 20 experiential exploratories of CCD-uniform precision with different combinations of the three independent factors along with experimental and theoretically predicted results of ALP production with residuals. In contrast, the other variable according to the PBD results exerted a negative effect on the ALP production process; hence, these insignificant variables were omitted from further optimization by uniform precision-CCD to improve enzyme production and diminish the final cost of the production process. The six axial points, eight factorial points, and six center points formed a precision random array for CCD in 20 empirical probationary for optimizing the picked variables. To assess the experimental errors, the six center point replicates were conducted. The findings indicated considerable variation in the potency of ALP activity depending on the three independent factor variations. ALP activities ranged from 935 to 5316.66 U L^−1^ based on the observed data. In Table 3, center point experimental trials (numbers 1, 4, 7–9, and 14) that comprise zero-level molasses, NaNO_3_, and MgCl_2_·6H_2_O concentrations (30, 0.6, and 0.45 g L^−1^, respectively) were recorded to have the highest ALP yield with an approximate activity of 5306.47 to 5316.66 U L^−1^. In contrast, the lowest ALP titer was achieved at the highest molasses (50 g L^−1^) concentration and middle NaNO_3_ (0.6 g L^−1^) and MgCl_2_·6H_2_O (0.45 g L^−1^) concentrations with an ALP activity of 935 U L^−1^. This resulted from the suppression effect of molasses, as reported by Qureshi et al.^19^. The theoretically forecasted ALP productivity was in line with the experimentally achieved ALP activity.

**Table 3:** Matrix design for *Lysinibacillus* sp. strain APSO CCD-uniform precision.

StdOrder	Trials	Type	Variables			ALP productivity (U L^−1^ min^−1^)		
			X_1_	X_2_	X_3_	Actual value	Predicted value	Residual
17	1	Center	0.000	0.000	0.000	5315.3023	5302.5	12.74796686
8	2	Factorial	1.000	1.000	1.000	1102.0370	806.44	295.5874
2	3	Factorial	1.000	− 1.000	− 1.000	1403.5185	1203.5	199.9446
18	4	Center	0.000	0.000	0.000	5316.6604	5302.5	14.10599
5	5	Factorial	− 1.000	− 1.000	1.000	1670.3703	930.84	739.5271
9	6	Axial	− 1.6817	0.000	0.000	1073.5185	1775.6	− 702.117
16	7	Center	0.000	0.000	0.000	5309.1912	5302.5	6.63686
15	8	Center	0.000	0.000	0.000	5306.4752	5302.5	3.92082
20	9	Center	0.000	0.000	0.000	5309.8702	5302.5	7.31587
14	10	Axial	0.000	0.000	1.6817	1464.6296	1747.07	− 282.447
3	11	Factorial	− 1.000	1.000	− 1.000	4406.5185	3758.03	648.4854
1	12	Factorial	− 1.000	− 1.000	− 1.000	1432.0370	1485.1	− 53.1299
13	13	Axial	0.000	0.000	− 1.6817	2851.8518	2912.2	− 60.4404
19	14	Center	0.000	0.000	0.000	5316.6604	5302.5	14.1059
11	15	Axial	0.000	− 1.6817	0.000	1360.7407	1529.44	− 168.708
4	16	Factorial	1.000	1.000	− 1.000	1140.7407	1637.81	− 497.069
7	17	Factorial	− 1.000	1.000	1.000	1994.2592	1951.74	42.5128
10	18	Axial	1.6817	0.000	0.000	935	575.770	359.229
6	19	Factorial	1.000	− 1.000	1.000	1218.1481	1624.17	− 406.027
12	20	Axial	0.000	1.6817	0.000	2578.8888	2753.06	− 174.179

Variables	Code	Coded and actual levels						
		−1.6817	−1	0	1	1.6817		
Molasses	X_1_	10	20	30	40	50		
NaNO_3_	X_2_	0.2	0.4	0.6	0.8	1		
MgCl_2_.6H_2_O	X_3_	0.15	0.3	0.45	0.6	0.75		

### Multiple regression analysis and ANOVA

Arithmetic operators of multiple regression statistical analysis and ANOVA computations, a critical approach to evaluating the significance and adequacy of the quadratic regression model, were employed to analyze and interpret the CCD-uniform precision experimental data results as enumerated in Table 4. In Table 4, the determination coefficient *R*^2^-value of this regression model was 0.964, indicating that 96.40% of the disparity in ALP productivity was attributed to the independent variables and only 3.60% of the total disparity created by variables could not be explained by the model and could not explain ALP activity. This regression model is trustworthy to reflect reality correlations between independent nutrimental variables that influence ALP throughput.

**Table 4:** Statistical analysis of CCD-uniform precision showing coefficient values, *t*-values, and *p*-values for each variable on ALP activity.

Term	Estimate	Std Error	*t*-Ratio	Prob >|*t*|	Confidence level (%)
Intercept	5302.554	194.5854	27.250	1.9264E^−08^	100
X_1_	− 356.7224	129.1031	− 2.7630	0.02002	97.9976
X_2_	363.78493	129.1031	2.8177	0.01822	98.1770
X_3_	− 346.42114	129.1031	− 2.683	0.02296	97.7037
X_1_*X_2_	− 459.65740	168.6812	− 2.7250	0.021375	97.8624
X_1_*X_3_	243.73148	168.6812	1.4449	0.17907	82.0926
X_2_*X_3_	− 312.99074	168.6812	− 1.8555	0.093196	90.6803
X_1_*X_1_	− 1459.0671	125.6786	− 11.609	3.98486E^−07^	99.9999
X_2_*X_2_	− 1117.6905	125.6786	− 8.8933	4.60585E^−06^	99.9995
X_3_*X_3_	− 1051.0717	125.6786	− 8.3632	7.96433E^−06^	99.9992

	df	SS	MS	*F*	Significance *F*
Regression	9	62,414,086	6,934,898	30.46608	4.15431E^−06^
Residual	10	2,276,268	227,626		
Total	19	64,690,354			
*R*^*2*^	0.964				
Adjusted *R*^*2*^	0.933				

Additionally, the model’s precision to calculate the predicted ALP output and a significant correlation between the observed and fitted values for ALP production was confirmed by the coefficient of multiple correlations (multiple *R*) of 0.982 and the adjusted determination coefficient value (adjusted *R*^2^) of 0.933 and predicted *R*^2^-value of 0.709. Furthermore, the model’s significance, fitness, and reliability determined by the low percentage of the coefficient of variation value (16.89%) and the root mean square error and mean values were 477 and 2825, respectively. Adequate precision measured the signal-to-noise ratio; a precision value of 14.008 indicated an adequate signal, and this model could be used to navigate the design space. ANOVA revealed the chosen model to be fabulously relevant, as obvious from the low probability *p* of 0.00000415 and the Fisher’s *F*-test of 30.4660, computed as the ratio of mean square regression and mean square residual. The higher the *F*-value is, the higher is the connotation of the model. *p*-values, *F*-values, and *t*-test were utilized for data interpretation, verification of the significance for each variable, and recognition of the mutual interaction dynamics between variables under investigation as illustrated in the fit summary results (Table 4). Furthermore, the interrelation between two variables can be either positive or negative to the response depending on the signs of the variable coefficients.

A negative sign implies an antagonistic effect, but the synergistic or complementary effect of two variables on the response is demonstrated by a positive sign. Accordingly, the antagonistic or oppositional effect (negative coefficients) was apparent in the mutual interaction between molasses (X_1_) and sodium nitrate (X_2_) together with the mutual interaction between sodium nitrate (X_2_) and MgCl_2_·6H_2_O (X_3_), with *F*-values (7.42 and 3.44, respectively), *p*-values (0.0214 and 0.0932, respectively), and *t*-values (− 2.7250 and − 1.8555, respectively) with confidence levels (97.8% and 90.6%, respectively). Based on the negative sign of a coefficient, these mutual interaction effects are nonsignificant model terms and do not improve the response. In contrast, mutual interaction between molasses (X_1_) and MgCl_2_·6H_2_O (X_3_) had a synergistic and significant effect on ALP productivity, obviously from the *F*-value of 2.09 and *t*-test value of 1.4449. Although its *p*-value and contribution percentage were 0.179 and 82%, respectively, it was a positive coefficient sign, contributing to the elevation and enhancement of ALP production. Another point worth mentioning is that the linear coefficients of sodium nitrate (X_2_) had highly significant effects on ALP productivity, as manifested from *p*-value (0.0182), *F*-value (7.94), and *t*-value (2.817) with confidence level (98.17%). Although the linear coefficients of molasses (X_1_) and MgCl_2_·6H_2_O (X_3_) together with the quadratic effects of molasses (X_1_^2^), NaNO_3_ (X_2_^2^), and MgCl_2_·6H_2_O (X_3_^2^) were characteristic of the negative sign of coefficients, they had not significantly contributed to the enhancement of ALP production by the strain under study. Arithmetic indications for their significance degrees were computed as follows: *p*-value (0.02, 0.022, 0.00000039, 0.0000046, and 0.0000079, respectively), *F*-value (7.63, 7.2, 134.7, 79, and 69.9, respectively), and *t*-test (− 2.76, − 2.68, − 11.6, − 8.89, and − 8.36, respectively) with confidence level (97.99%, 97.7%, 99.99%, 99.99%, and 99.99%, respectively). The second-order polynomial function fitted to the experimental results of ALP productivity was employed for predicting the optimal point within experimental constraints as in the following equation:

$${\text{Y}} = 5302.55 - 356.72{\text{X}}_{1} + 363.78{\text{X}}_{2} - 346.42{\text{X}}_{3} - 459.65{\text{X}}_{1} {\text{X}}_{2} + 243.73{\text{X}}_{1} {\text{X}}_{3} - 312.99{\text{X}}_{2} {\text{X}}_{3} - 1459{\text{X}}_{1}^{2} - 1117.69{\text{X}}_{2}^{2} - 1051.07{\text{X}}_{3}^{2}$$

where Y is the predicted response (ALP activity), and X_1_, X_2_, and X_3_ are the coded levels of the independent variables of molasses, NaNO_3_, and MgCl_2_·6H_2_O, respectively.

### Model adequacy checking

**Figure 4:**
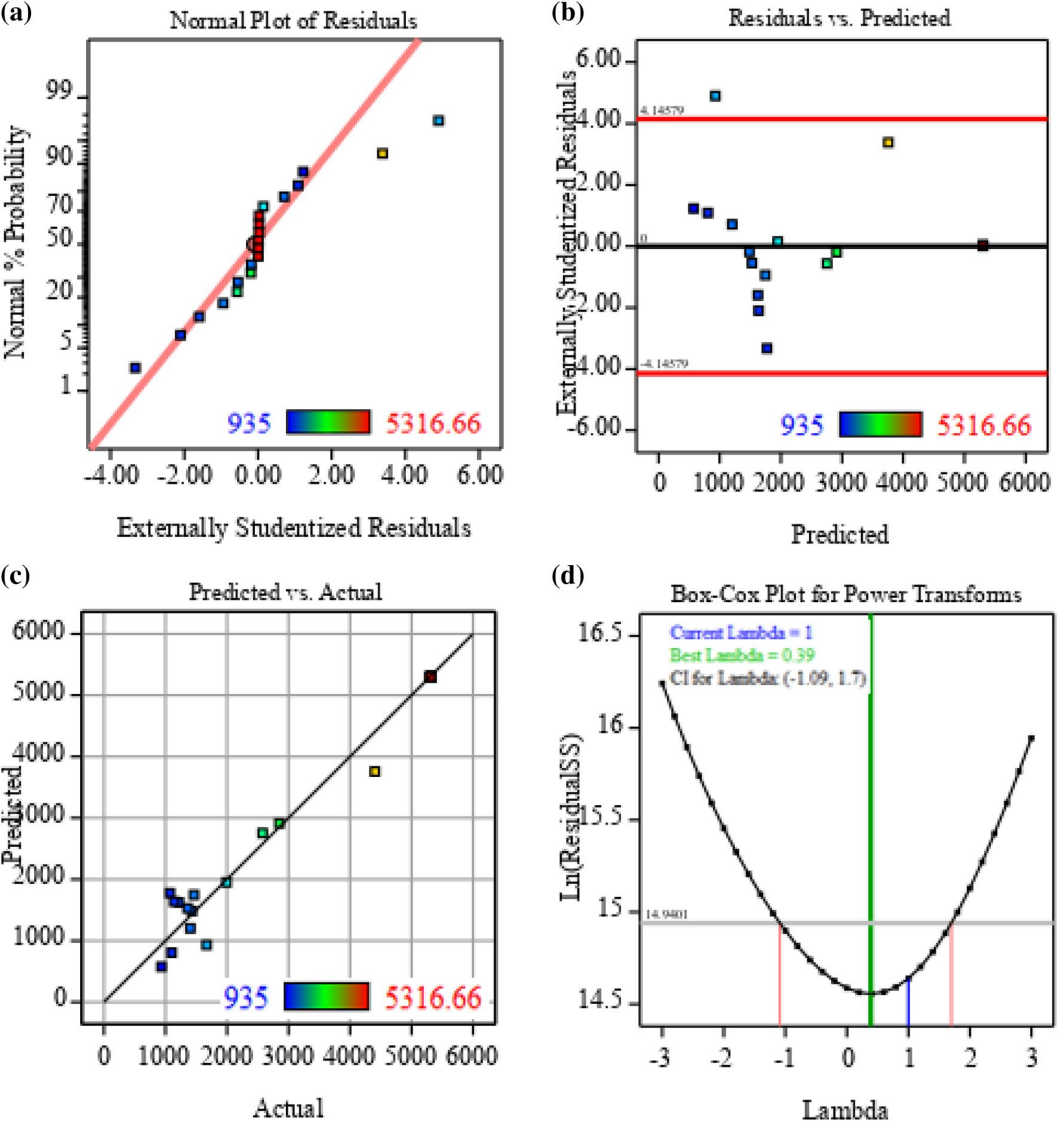
Model adequacy checking of CCD-uniform precision: **(a)** normal probability plot of the residuals, **(b)** externally studentized residuals versus predicted ALP production, **(c)** plot of predicted versus actual ALP production, and **(d)** Box-Cox plot of model transformation.

Some empirical statistics were conducted to ensure that the model was appropriate to meet the assumptions of the analysis. To illustrate whether the residuals follow a normal distribution or deviated from it, the distribution residuals were drawn against a theoretical normal distribution through the normal probability plot. As shown in the normal probability plot of the studentized residuals in Fig. 4a, the plotted points are located nearly to a straight line without linearity, indicating that the model has been well fitted with the experimental results. In contrast, most of the findings clustered in the center and the values distant from the general mean dwindled on both sides of the center peak equally. Extreme residual values are not preferred on both sides. Additionally, the residual versus predicted response chart was constructed and is depicted in Fig. 4b, through which randomly scattered residuals around the horizontal zero-line reference were observed and no particular patterns could be identified. In essence, this implied that the residuals are not correlated and distributed independently and thus have constant variance, meaning that the model is adequately compliant with the study’s postulations. This indicated a good fit of the model for ALP production by *Lysinibacillus* sp. APSO. Moreover, plotting the predicted values against the actual data sets (Fig. 4c) revealed that the data sets were divided evenly down the 45° parallel. This could help detect if the model cannot smoothly predict some value(s); however, all data points are measurable, guaranteeing model aptness. Figure 4d clarifies the Box-Cox graph. The best λ-value (− 0.39) was represented by the green line, and this transformation (λ = 1) was denoted by the blue line, whereas the red lines symbolized the lowest and highest values of confidence intervals between − 1.09 and 1.7. Because the λ best value was between the values of confidence intervals, no recommendation for data transformation was required due to the blue line between the two red lines. This revealed that the model was well appropriate to the obtained experimental data.

### Contour and three-dimensional (3D) plots

**Figure 5:**
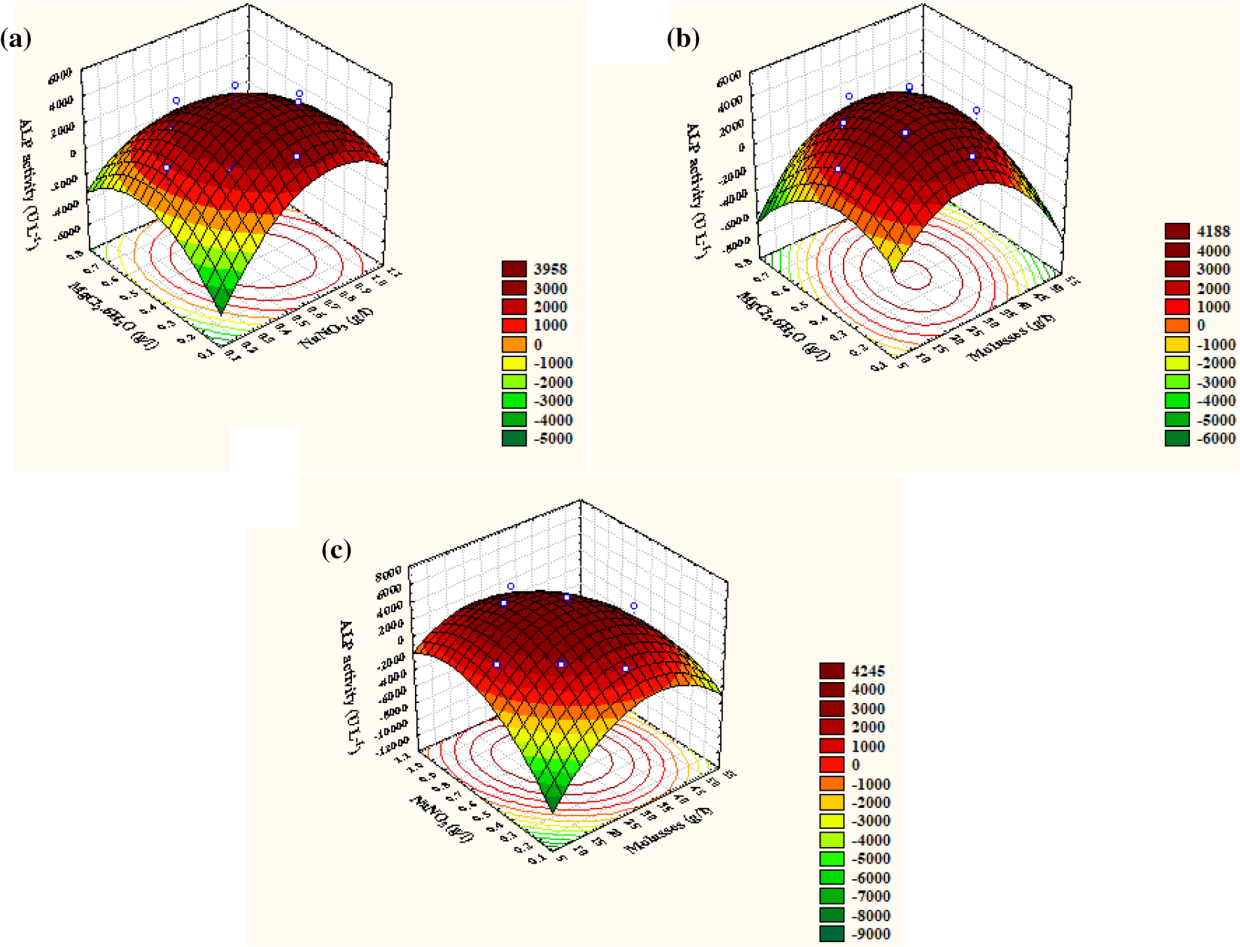
3D response surface representing ALP activity yield (U L^−1^ min^−1^) from *Lysinibacillus* sp. strain APSO as affected by culture conditions: **(a)** interaction between MgCl_2_·6H_2_O and NaNO_3_, **(b)** interaction between molasses and MgCl_2_·6H_2_O, and **(c)** interaction between molasses and NaNO_3_.

After the preceding assurance of model appropriateness, the interactive effects of the three measured variables were defined. Also, the optimal values of each independent variable needed for maximal ALP production were calculated through the construction and visualization of 3D surface and outline contour chart by plotting ALP activity on the Z-axis against two of the variables while keeping the third variable at the center point, as shown in Fig. 5.

The 3D surface and contour plot of Fig. 5a shows ALP productivity efficacy as a function of molasses (X_1_) and NaNO_3_ (X_2_) concentrations with maintained MgCl_2_·6H_2_O concentration at zero levels (0.45 g L^−1^). The highest point for ALP activity was situated around the center points of molasses, out of this range; rather, low ALP productivity was noticed. Further, ALP activity was enhanced with increments of NaNO_3_ concentrations until reaching its optimum, but the higher level supporting low ALP activity was remarked. The maximal predicted pinpoint of ALP output was estimated through the software program of the point prediction option. The maximal predicted ALP activity of 5363.26 U L^−1^ was attained at the optimal predicted molasses (28.23 g L^−1^) and NaNO_3_ (0.645 g L^−1^) concentrations at a MgCl_2_·6H_2_O concentration of 0.45 g L^−1^. The same trend of ALP productiveness was noticed for the pairwise combination of molasses (X_1_) and MgCl_2_·6H_2_O (X_3_) with zero levels of NaNO_3_ (X_2_) 0.6 g L^−1^. In Fig. 5b, the highest ALP throughput was recorded at a low MgCl_2_·6H_2_O (0.4 g L^−1^) concentration near the middle point; the molasses concentration increased, resulting in a declining trend of ALP productivity. Beyond the optimal center point of X_1_ and X_3_, their lower and higher levels caused a relative increase in ALP yield. Therefore, by solving Eq. (3) and analyzing Fig. 5b, using a zero-level NaNO_3_ (0.6 g L^−1^) concentration at the predicted optimal molasses (28.23 g L^−1^) and MgCl_2_·6H_2_O (0.41 g L^−1^) concentrations contributed to the utmost ALP predicted productivity of 5354.8 U L^−1^. The 3D plot and the corresponding contour plot (Fig. 5c) highlighted the effects of NaNO_3_ (X_2_) and MgCl_2_·6H_2_O (X_3_) concentrations on ALP production at 30 g L^−1^ of zero-level molasses (X_1_) concentration. ALP activity increased progressively at a low NaNO_3_ concentration, accompanied by increasing MgCl_2_·6H_2_O concentrations to its optimum; then, ALP production declined. The greatest ALP throughput was clearly supported at the center level of MgCl_2_·6H_2_O (X_3_), passing the optimal points of its concentration; whether an increase or decrease, it will lead to a reduction in productivity. By solving Eq. (3) and analyzing Fig. 5c, the maximal predicted ALP activity of 5368.31 U L^−1^ was attained at the optimal predicted NaNO_3_ (0.64 g L^−1^) and MgCl_2_·6H_2_O (0.41 g L^−1^) concentrations at a molasses concentration of 30 g L^−1^.

### Verification of the experimental model

To estimate the optimal combinations of the variables under investigation, which ameliorate ALP productivity, response optimization was carried out to augment the efficacy of *Lysinibacillus* sp. APSO ALP. The optimal predicted molasses (28.23 g L^−1^), NaNO_3_ (0.64 g L^−1^), and MgCl_2_·6H_2_O (0.41 g L^−1^) concentrations for boosting the efficacy of ALP production in the fermentation medium to 5413.91 U L^−1^ were predictable and theoretically based on the equation of the quadratic model. Laboratory validation via bench-scale experiments was utilized to confirm the theoretical data results of the optimization process, revealing that the practical ALP activity of 5683 U L^−1^ was near that obtained theoretically by the regression equation of the model (5413.91 U L^−1^) with 104.97% accuracy, confirming the model’s authenticity. Hence, the ultimate culture conditions and medium composition were molasses, 28.23 g L^−1^; NaNO_3_, 0.64 g L^−1^; and MgCl_2_·6H_2_O, 0.41 g L^−1^, and the pH was adjusted to 7.0 by 1 M NaOH with 1% old activated preculture inoculum. The inoculated medium was incubated at 45 °C and 200 rpm for 24 h.

Compared to the results cited by other investigators, very few investigators dealt with statistical optimization strategies for improving ALP productivity. Pandey et al.^21,25^ were the only researchers who exploited CCD to enhance the production process of ALP from *B. licheniformis*. In 2010^21^, they documented the optimization strategies of physical variables that influence ALP productivity. Their study revealed that the maximal ALP yield of 792.043 U mL^−1^ was achieved at pH 8.0, temperature of 36.7 °C, fermentation time of 78 h, and orbital speed of 165 rpm. In 2012^25^, the same team recorded that the predicted glucose (2.39%), peptone (1.35%), yeast extract (0.15%) concentrations were obtained from the theoretical calculation of the regression model of CCD for enhancing the *B. licheniformis* ALP output. They documented that the correlation coefficient (*R*^2^) was found to be 0.932 (93.2%). Recently, through the regression model of rotatable-CCD by Abdelgalil et al.^16^, the highest predicted ALP productivity (3753.27 U L^−1^) produced from *B. paralicheniformis* strain APSO could be attained using molasses, (NH_4_)_2_NO_3_, and KCl concentrations of 30, 0.79, and 1.2 g L^−1^, respectively.

### Scale-up fermentation strategies for *Lysinibacillus* sp. APSO ALP

The improvement of ALP productivity is needed, and the biomass necessary for sufficient yield is economically limited unless the phosphatase-specific activity is augmented. Therefore, the opportunity for optimizing growth conditions, which give the highest specific phosphatase activity, was taken^26^.

### Cell growth kinetics and ALP production in shake-flask under batch conditions

**Figure 6:**
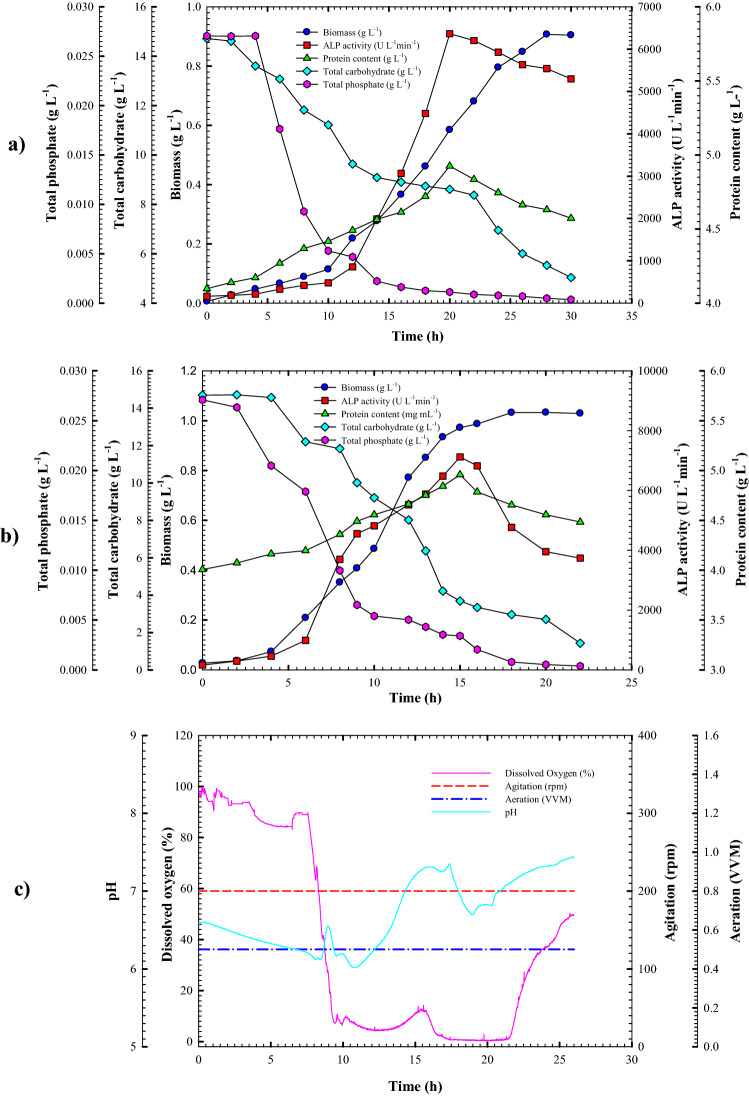
Monitoring of *Lysinibacillus* sp. strain APSO growth and ALP productivity in **(a)** a shake-flask scale cultivation condition and **(b)** a 7 L stirred-tank bioreactor under uncontrolled pH conditions. **(c)** Online data (DO, agitation, airflow rate, and pH) as a function of time during batch fermentation in the bioreactor under uncontrolled pH conditions.

Before moving forward to bioreactor cultivations, this study intended to determine the correlations between the specific growth rate of the culture and the ALP production rate via a cell growth kinetics study in a shake-flask cultivation system, which could likely be referred to for the bioreactor strategy for batch cultivation. Table 5 illustrates the cell growth kinetics and ALP production parameters by *Lysinibacillus* sp. strain APSO affected by different cultivation strategies. Batch growth kinetics of *Lysinibacillus* sp. strain APSO followed a typical growth pattern, and ALP was produced simultaneously with cell growth, as shown in Fig. 6a. This finding was analogous to Butler et al.^27^ and Abdelgalil et al.^16^, who found that phosphatase production increased during exponential growth of a *Citrobacter* sp. and *B. paralicheniformis* strain APSO, respectively.

**Table 5:** Cell growth kinetics and ALP production parameters by *Lysinibacillus* sp. strain APSO as affected by different cultivation strategies.

Parameters	Shake flask cultivation	Uncontrollable pH Batch Cultivation	Controllable pH Batch Cultivation
**Growth parameters**			
X_max-conc._ (g L^−1^)	0.90666	1.03223	0.70292
µ (h^−1^)	0.115	0.18875	0.127883
µMax (h^−1^)	0.776909	0.52260	0.4633217
dX/dt (g L^−1^ h^−1^)	0.0440305	0.068482	0.035143
**Production parameters**			
P_max_ (U L^−1^)	6365.74	7119.44	5795.37
P_max.specific_ (U g^−1^)	1292.4222	1435.889	1215.026
P_max.time_ (h)	20	15	20
Q_p_ (U L^−1^ h^−1^)	588.703	605.5555	387.764
-Q_s_ (g L^−1^ h^−1^)	0.26068	0.989393	0.559922
-Q_s_ (g L^−1^ h^−1^)	0.0004169	0.0017068	0.000805
**Yield coefficient parameters**			
Y_p/s_ (U g^−1^)	965.85	1123.62	639.93
Y_x/s_ (g g^−1^)	0.07	0.11	0.04
Overall cultivation time (h)	30	22	26

*X*_*max-conc*_ maximal cell dry weight, *dx/dt* cell growth rate, *µ* specific growth rate, *P*_*max*_ maximal ALP production, *P*_*max,specific*_ specific productivity, *Q*_*p*_ ALP production rate, *Q*_*s*_ substrate consumption rate, *Y*_*p/x*_ (*U* *g*^*−1*^) ALP produced/g biomass, *Y*_*p/s*_ (*U* *g*^*−1*^) ALP produced/g substrate consumed, *Y*_*x/s*_ *(g* *g*^*−1*^*)* biomass produced/g substrate consumed.

Initial lag-phase cells experienced exponential growth with a growth rate of 0.044 (g L^−1^ h^−1^) and a specific growth rate (µ) of 0.115 h^−1^. The culmination of biomass production (0.906 g L^−1^) was achieved at 28 h cultivation period with an obvious yield coefficient Y_x/s_ (0.07 g of cells/g of the substrate), surpassing this point, driving cell growth to stationary phase. The volumetric ALP production and protein content were concurrent with the cell growth pattern. Both increased gradually until reaching their zenith (6365.74 U L^−1^ and 4.92 g L^−1^, respectively) with production rate Q_p_ (588.703 U L^−1^ h^−1^), specific productivity P_max,specific_ (1292 U g^−1^), and yield coefficient Y_p/s_ (965.85 U g^−1^) at 20 h incubation time. A gradual decline in ALP activity and protein content was observed beyond this time. Meanwhile, due to cell growth, proliferation, and the essential role of ALP in phosphate transportation and metabolism^25^, a notable decrease in total carbohydrate and total phosphatase concentrations was found from their initial concentrations of 14.71 and 0.027 g L^−1^ to reach 5.03 and 0.0003 g L^−1^, with a consumption rate of − 0.26068 and 0.0004169 g L^−1^ h^−1^, respectively. From the pH profile throughout the fermentation process (Fig. 6a), at the first 10 h incubation period, considered a lag-phase period, a gradual elevation in the initial pH of the culture medium from 6.4 to 7.6 occurred but decreased to 7.0 at 18 h. After that time, the pH increased again with up-down fluctuations to reach 8.0 at 30 h cultivation time. This pH change was accompanied by a gradual increase of bacterial growth, ALP productivity, and protein content to reach their own climax. This resulted from the influence of pH on bacterial growth or pH-dependent control of the gene expression for enzyme synthesis^19^.

There was an obvious need for metal ions to promote ALP productivity. Chaudhuri et al.^2^ reported that the regulatory control was mainly carried out by metal ion chelation necessary for enzyme production or stabilization. As noticed from the heavy metal survey throughout the cultivation period via atomic absorption spectrometry (AAS) analysis, the concentration of some metal ions, such as Cu^+^, Pb^+^, Fe^+^, Zn^+^, Mg^+^, Sn^+^, and Ni^+^, was synchronous with ALP productivity. In contrast, the apex of their concentrations (4.04, 20.36, 51.1, 5.835, 1530, 287.9, and 5.25 mg L^−1^) was achieved when the peak of ALP production was obtained, which was 20 h incubation time. After transcending this point, their concentrations decreased until the end of the fermentation period, as shown in Table 6. Li^+^ reached its peak (1.27 mg L^−1^) at the end of cultivation time after up-down fluctuations throughout the fermentation period, there was no change observed in Co^+^, Cr^+^, and Mn^+^ concentrations throughout the fermentation period, and Cd^2+^ ions were consumed early to < 0.0335 mg L^−1^ during the growth course. This finding was in agreement with that recorded by Abdelgalil et al.^16^, who noticed that the growth of *B. paralicheniformis* strain APSO was associated with an increment in Sn^+^, Zn^+^, Mg^+^, and Mn^+^ ion concentrations, reaching their peak of 283, 22, 652.5, and 4.27 mg L^−1^ at 26 h, at the peak of ALP output. There was no change in Co^+^ and Cr^+^ concentrations throughout the fermentation period.

**Table 6:** Flame AAS for heavy metal analysis of shake-flask batch cultivation.

Metal ions (mg L^−1^)	Incubation time (h)					
	0	6	10	14	20	30
Co	< 0.0982	< 0.0982	< 0.0982	< 0.0982	< 0.0982	< 0.0982
Cu	0.997	0.75	1.0325	1.637	4.04	0.9075
Pb	< 0.2696	< 0.2696	< 0.2696	< 0.2696	20.36	< 0.2696
Fe	8.392	24.25	10.8	24.155	51.1	22.26
Ni	< 0.3662	5.712	< 0.3662	4.34	5.25	1.5625
Cd	0.05	< 0.0335	< 0.0335	< 0.0335	< 0.0335	< 0.0335
Zn	2.16	3.18	3.756	4.695	5.835	2.3825
Mg	630	543.75	596.25	517.5	1530	1046.25
Mn	< 0.0627	< 0.0627	< 0.0627	< 0.0627	< 0.0627	< 0.0627
Li	0.3275	0.782	0.395	0.426	0.865	1.27
Sn	72	38.75	139.4	174.62	287.9	147.3
Cr	< 0.7276	< 0.7276	< 0.7276	< 0.7276	< 0.7276	< 0.7276

### Cell growth kinetics and ALP production in the bioreactor under uncontrolled pH batch conditions

The scale-up strategy is required to reproduce the culture characteristics and kinetic parameters from shake-flasks to fermenters. Therefore, the cultivation kinetics of *Lysinibacillus* sp. strain APSO was monitored in a 7 L stirred-tank bench-top bioreactor (Bioflo 310; New Brunswick Scientific, Edison, NJ, USA) under uncontrolled pH for further development and optimization. Knowledge of the fermentation process kinetics may be valuable for improving the performance of batch processes. Finally, product yield and substrate conversion are the most significant criteria toward productivity^28^. The results showed great similarity in the growth and ALP production pattern to those achieved in the shake-flask counterpart (Fig. 6b).

A significant improvement was seen in cell growth and enzyme production under uncontrolled pH batch cultivation conditions. The peak of cell biomass production (1.0322 g L^−1^) was achieved at 18 h incubation time, earlier than those in shake-flasks by ~ 10 h, whereas the stationary phase started beyond this point at 20 h incubation time. Cells growing exponentially with a growth rate of 0.068482 g L^−1^ h^−1^ and a specific growth rate (µ) of 0.18875 h^−1^ showed a yield coefficient Y_x/s_ of 0.11 g cells/g substrate), considerably higher than those achieved by shake-flask cultivation. Further, ALP production and protein content were simultaneous with the growth pattern. The peak volumetric ALP productivity was accomplished at 15 h cultivation with an activity of 7119.44 U L^−1^ and a production rate Q_p_ of 605.55 U L^−1^ h^−1^, which were about 111.83% and 102.86%, respectively, higher and earlier than those in shake-flask cultivation by ~ 5 h. Moreover, considerable elevation in ALP yield coefficient Y_p/s_ of 1123.62 U g^−1^ was noticed, about 1.16 times higher than the obtained counterpart. It is noteworthy that growth and ALP production patterns were accompanied by substrate consumption patterns. With cell growth and enzyme productivity increased progressively, substrate consumption also increased, leading to a trend of total carbohydrate and total phosphate concentrations decreasing from their initial concentrations (14.71 and 0.027 g L^−1^, respectively) to reach their minimal concentrations (1.42 and 0.00038 g L^−1^, respectively) at the end of the cultivation period. This was accompanied by an increasing consumption rate of total carbohydrate and total phosphate (− 0.98 and − 0.0017 g L^−1^ h^−1^, respectively) by > 3.79- and 4.1-fold, respectively, than those in shake-flask cultivation. Compared to the findings of Abdelgalil et al.^16^, ALP throughput of 6650.9 U L^−1^ and µ of 0.0943 h^−1^ were achieved at 8 h incubation time of *B. paralicheniformis* strain APSO under uncontrolled pH batch cultivation conditions in 7 L stirred-tank bioreactor.

Gupta et al.^29^ reported that changes in oxygen availability might lead to drastic effects on fermentation kinetics. Therefore, in this investigation, dissolved oxygen (DO) availability in the culture medium was monitored throughout the cultivation period. Figure 6c shows that the DO percentage at lag phase times of 2 and 4 h was 100% and 96%, respectively. Once bacterial cells moved toward the exponential phase, the DO percentage declined sharply, reaching 0.7% at the mid-exponential phase (10 h) due to actively growing cells. After this point, it was at a steady state from 12 to 20% until 15 h. This was synchronous with ALP peak production. At the late log-phase and the beginning of the stationary phase, it increased again in obvious increments, reaching 88.9% at the end of the fermentation period. Additionally, the tracking of the culture medium pH showed the same pattern as those in the shake-flask counterpart. A noticeable decline in pH values from 6.45 to 5.96 was noticed at the initial lag-phase period from incubation time. After 6 h, a gradual increase in the culture medium pH was observed, reaching 8.07 at the end of the fermentation period. This matched that of previous shake-flask cultivation.

Based on the above results, a > 111.83% improvement of peak volumetric productivity was achieved, higher than in shake-flask cultivation (6365.74 U L^−1^ at 20 h). These enhancements in efficiency resulted from good cultivation of oxygenation and involvement conditions and the vessel’s bioreactor capacity. However, maximal volumetric productivity increased by 148.18% from that in shake-flask cultivation (4488.266 U L^−1^ at 26 h) by *B. paralicheniformis* strain APSO^15^.

### Cell growth kinetics and ALP production in the bioreactor under controlled pH batch conditions

**Figure 7:**
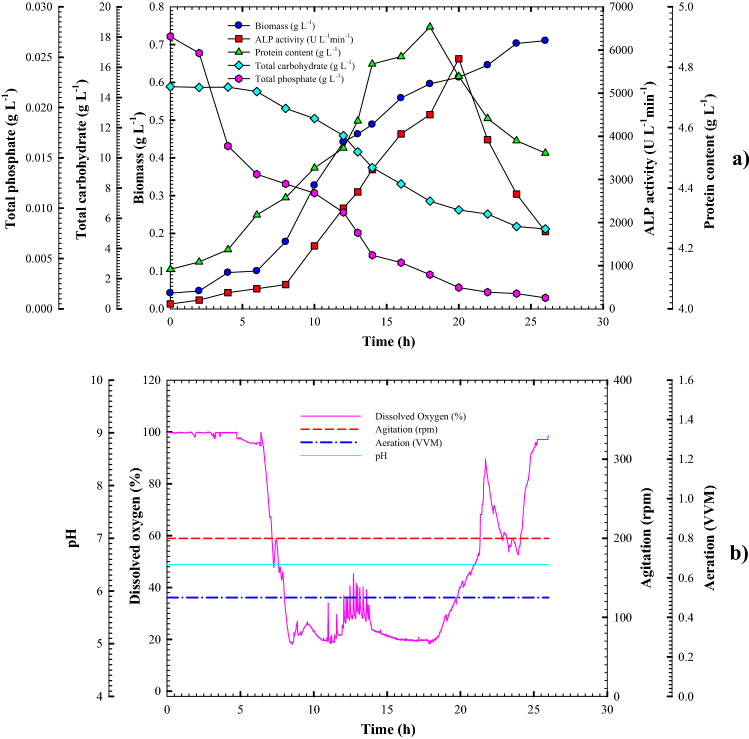
**(a)** Monitoring of *Lysinibacillus* sp. strain APSO growth and ALP productivity in a 7 L stirred-tank bioreactor under controlled pH conditions. **(b)** Online data (DO, agitation, airflow rate, and pH) as a function of time during said batch fermentation.

Based on previous results, uncontrolled pH batch cultivation showed the best conditions for improving and enhancing volumetric and specific ALP productivity. In this study, *Lysinibacillus* sp. strain APSO was forced to grow under controlled pH culture conditions (pH 6.45) through a batch cultivation system in a 7 L stirred-tank bench-top bioreactor (Bioflo 310) to evaluate the influence of constant pH value on the growth and productivity characteristic features. Figure 7a shows the relations between cell growth, enzyme production, and substrate consumption as a function of time for batch fermentation of *Lysinibacillus* sp. strain APSO under controlled pH conditions. In Fig. 7a, the general patterns show an analogy with those in shake-flask cultivation. As pointed out in previous cultivation conditions, the ascending increment of cell growth was noticed at 6 h cultivation period with a growth rate of 0.035143 g L^−1^ h^−1^ until reaching its peak (X_max_ = 0.702 g L^−1^) at 24 h, about 22.51% and 31.9% lower than that in shake-flask and uncontrolled pH batch cultivation, respectively. Furthermore, the lowest yield coefficient Y_x/s_ (0.04 g cells/g substrate) was obtained compared to previous culture conditions.

Additionally, volumetric enzyme productivity and protein content patterns were parallel to cell growth patterns, with a lesser production rate Q_p_ of 387.764 U L^−1^ h^−1^ than those in previous batch cultivations by 34.13% and 35.96%, respectively. The targeted productivity peak (5795.37 U L^−1^) was achieved at 20 h after such a point the production curve started to decrease, about 8.95% and 18.59% lesser than those achieved in shake-flask and uncontrolled pH batch cultivation, respectively. However, a 33.7% to 43.04% decline in yield coefficient Y_p/s_ (639.93 U g^−1^) than those in other counterparts was noticed. Also, protein content decreased rapidly after overstepping its peak point (4.93363 g L^−1^) at 18 h. The correlation of substrate consumption with the growth and enzyme production pattern led to a decline in total carbohydrate and total phosphate concentrations throughout the fermentation period, reaching 5.292 and 0.001 g L^−1^ at the end of cultivation (26 h) with a consumption rate of − 0.559 and − 0.0008 g L^−1^ h^−1^, respectively. The consumption rate indicated the lower substrate consumption than consumed during the uncontrolled pH batch cultivation system. This resulted from a reduction in cell growth and enzyme productivity. Moreover, a slight elevation in DO concentration (100%) was observed during the first 4 h. With cell growth, DO consumption increased, leading to a decline in DO concentration to 19.2% at 16 h. Then, it increased again until the end of the cultivation time to reach 85% (Fig. 7b).

With bacterial cells growing and surviving under controlled pH conditions, unfavorable results were obtained because a steady pH hindered cell growth and adaption. Under these conditions, *Lysinibacillus* sp. strain APSO lost its ability to produce ALP with efficiency. Therefore, the uncontrolled pH culture condition represents the most suitable and favored condition for supporting and enhancing ALP productivity. This finding was congruent with Abdelgalil et al.^16^, who noticed that controlled pH conditions neither supported cell growth nor enhanced ALP productivity.

The sequential optimization strategy led to a systematic improvement of *Lysinibacillus* sp. strain APSO ALP productivity, which started with PBD (3208.33 U L^−1^), followed by CCD-uniform precision (5683 U L^−1^), and ended with uncontrolled pH batch cultivation strategy designs (7119.44 U L^−1^). Throughout the process, productivity was enhanced by 9.3-, 16.5-, and 20.75-fold, respectively, compared to basal medium (343.44 U L^−1^).

Due to the scarcity of specific literature, a comparison of results to others is difficult. This study is the first to report the scale-up production of extracellular ALP from *Lysinibacillus* sp. strain APSO on a bench-top bioreactor scale using sugarcane molasses as a nutrient source for stimulating ALP production.

## Conclusions

This study highlighted robust microbial engineering strategies for the large-scale production of newfound ALP by a local bacterial isolate. Therefore, the most potent ALP-producing bacterial isolate from a slime sample from Alexandria Paper and Pulp Factory was used as a model and identified as *Lysinibacillus* sp. strain APSO through molecular and morphological characterization. To reduce the cost of the ALP production process, an industrial by-product of molasses was utilized as a carbon and enzyme stimulate. The efficiency of ALP productivity was achieved through sequential statistical optimization strategies, followed by bioprocessing scale-up approaches in bench-top bioreactors. A > 9.5-fold improvement in the efficiency of ALP yield was achieved by PBD compared to the initial basal medium. The results revealed that molasses, NaNO_3_, and MgCl_2_·6H_2_O are the most noteworthy variables that positively influence ALP throughput, so they were used for further enhancement via CCD-uniform precision to determine the correlation among the most important variables that provide superior throughput via a polynomial quadratic model. The noticeable enhancement in ALP productivity was attained at 16.5-fold increments compared to the initial basal medium, emphasizing sequential statistical optimization strategies for improving the production process. Mathematical models are kinetic models that describe the correlation between the rate and reactant/product concentration and allow to predict the product conversion rate. Hence, additional improvements were followed in a 7 L bench-top bioreactor to evaluate microbial growth kinetics in a closed cultivation system under controlled and uncontrolled pH conditions. ALP production was synchronous with the growth pattern and reached its peak in the early exponential phase of *Lysinibacillus* sp. strain APSO. The uncontrolled pH condition was supported and facilitated growth and enzyme production, whereas the pH-controlled system neither supported *Lysinibacillus* sp. strain APSO growth nor enhanced ALP productivity.

## Materials and methods

### Isolation and maintenance of ALP-producing bacteria

The slime samples assembled aseptically at Alexandria paper and pulp factory were utilized for isolating bacterial isolates throughout this study. The modified PVK broth medium [glucose, 10 g L^−1^; (NH_4_)_2_SO_2_, 0.5 g L^−1^; NaCl, 0.5 g L^−1^; yeast extract, 0.5 g L^−1^; KCl, 0.2 g L^−1^; MgSO_4_·7H_2_O, 0.1 g L^−1^; MnSO_4_·H_2_O, 0.002 g L^−1^; FeSO_4_·7H_2_O, 0.002 g L^−1^; eggshell powder, 5 g L^−1^, instead of Ca_3_(PO_4_)_2_; pH 7.0] was used for the enrichment and isolation of ALP-producing bacteria^11^. Eggs were purchased from the market, and their shells were collected, washed, and dried in an oven overnight at 60 °C. Eggshell were ground by blender and passed through a 0.125 mm sieve to obtain a fine eggshell powder.

A 50 mL modified PVK broth medium^11^ dispensed in 250 mL Erlenmeyer flasks was inoculated with a 1.0 g slime sample and incubated in a reciprocal shaker incubator (200 rpm) at 30 °C for 72 h. Afterward, a 50 µL aliquot of a 10^−6^ dilution sample, obtained by standard serial dilutions of the enriched cultivated samples using sterile water saline (0.85% NaCl, 0.1% peptone) as a diluent, was spread onto the surface of an LB agar plate using the spread plate method and incubated in a static incubator overnight at 30 °C. Discrete colonies were picked up under aseptic conditions for further purification by the streak plate method using a sterile LB agar plate. On LB slants, pure isolates were subcultured, kept at 4 °C, and regularly subcultured. Growing cells of pure isolates were maintained on 50% glycerol in cryogenic vials at − 80 °C for a master cell bank preservation.

### Qualitative screening for ALP activity

Two types of medium [MG-PDP medium: glucose, 1 g L^−1^; peptone, 10 g L^−1^; NaCl, 10 g L^−1^; yeast extract, 5 g L^−1^; and bacteriological agar, 20 g L^−1^, supplemented with 50 µg/mL MG dye and 1 mg/mL PDP^30^ and rich medium: glucose, 1 g L^−1^; peptone, 10 g L^−1^; NaCl, 10 g L^−1^; yeast extract, 5 g L^−1^; and bacteriological agar, 20 g L^−1^, supplemented with 1 mg/mL chromogenic substrate (ρNPP)^31^] were used as a screening medium for qualitative screening. Upon performing quantitative screening, a loopful of a pure colony of isolates was spot-inoculated on the middle of a screening medium plate. After overnight incubation of the inoculated plates at 30 °C, distinct yellow and/or green colonies appeared due to ALP production. APSO was designated as a profitable isolate with the highest color intensity and was picked out for further study.

### Quantitative assessment

Enzyme production was evaluated in submerged cultures of the most potential isolate in a modified PVK broth medium^11^ supplemented with 5 g L^−1^ eggshell powder. Upon performing quantitative screening, the medium was inoculated with 2% of 12 h activated old preculture of the promising isolate and incubated in a rotary shaker incubator (200 rpm) at 30 °C for 72 h. The supernatant with soluble target enzyme was obtained by centrifugation of the bacterial culture cellular pellet at 4 °C, 6000 rpm for 15 min and used to estimate ALP activity. Spectrophotometric assays for estimating ALP activity were conducted by following the absorbance change at 405 nm with a molar extinction coefficient (ε) of 18,000 M^−1^ cm^−1^ accompanying the hydrolysis of the chromogenic substrate (ρNPP) into ρ-nitrophenol, as reported by Obidi et al.^32^. For the standard assay, ρNPP was added to the reaction mixture of 0.1 M modified universal buffer pH 10.0 to a final concentration of 10 mM. The reaction was initiated by adding an appropriately diluted enzyme solution to the reaction mixture, followed by incubation at 65 °C for 3 min. Afterward, 0.05 mL of 4.0 M NaOH was added to terminate the reaction. The linear change in the light absorption of the product ρ-nitrophenol was monitored by an ultraviolet–visible (UV–vis) spectrophotometer at 405 nm. The spectrophotometer used a blank ρNPP_zero_ sample that contained the same mixture reaction constituents except the enzyme sample. An international unit (IU) of enzyme activity was defined as the quantity of enzyme catalyzing the liberation of 1.0 µM ρ-nitrophenol from ρNPP in 1 min at pH 10.0 and 65 °C; the activities were expressed in U L^−1^ min^−1^.

### DNA extraction, polymerase chain reaction (PCR) amplification, and sequence data analysis

The total genomic DNA of the overnight grown promising bacterial isolate was extracted using the methodology of Marahatta et al.^33^. PCR and sequencing were carried out in accordance with the method of Abdelgalil et al.^34^.

### Morphological characterization of *Lysinibacillus* sp. strain APSO

To observe the morphology of the strain under investigation, bacterial cells were harvested at the log-phase growth pattern during submerged cultivation, washed twice, and suspended in physiological saline. The dried bacterial thin film was coated with a thin layer of gold using a sputtering device (JFC-1100 E; Jeol; USA) for 12 min. A micrograph at 20 kV was obtained using a JSM 5300 (Jeol) scanning electron microscope (SEM) at the Laboratory Centre-City for Scientific Research and Technological Applications (SRTA-City).

### Effects of physical parameters on ALP productivity

Various physical parameters influencing ALP yield, such as incubation temperature, pH, and initial inoculum size, were studied individually. A 2% inoculum of the activated old preculture of the promising strain was aerobically cultured in 250 mL Erlenmeyer flasks containing 50 mL modified PVK broth medium at varying temperatures viz. 30 °C, 37 °C, 40 °C, and 45 °C under shaking conditions (200 rpm) to investigate the optimal temperature for ALP output. The determination of enzyme activity was carried out after 72 h incubation. To determine the effects of pH values on ALP productivity, the initial pH of the 50 mL modified PVK broth medium^11^ dispensed individually in 250 mL Erlenmeyer flasks was adjusted to 6.0, 7.0, 8.0, 9.0, and 10.0 using 0.1 M HCl and 0.1 M NaOH. Subsequently, flasks of the pH-adjusted medium were inoculated with a standard inoculum of activated preculture (2%) and incubated at the optimal temperature (45 °C) in a shaker incubator (200 rpm) for 72 h. To measure ALP activity, 2.0 mL samples were used.

Inoculum of different sizes (2%, 5%, and 10%) of the activated preculture of the isolate was used for the inoculation of 50 mL modified PVK broth medium to investigate the effect of inoculum size on ALP productivity after incubation of inoculated flasks at 45 °C in a rotary shaker incubator (200 rpm) for 72 h. ALP activity was measured in the drawn-out sample.

### Cost-effective optimization strategies for high ALP production

The implementation of mathematical designs to optimize the constituents of the medium during the fermentation process will fix the shortcomings of the traditional OFAT approach and can be an effective tool to enhance metabolite productivity^6^.

### PBD

Hadamard matrix designs are a particular form of two-level fractionate factorial designs (resolution III) developed by Plackett and Burman, which explores the effects of various parameters on a particular production process and prevents unwanted repetitions utilizing just a limited number of experiments^35^. The purpose of PBD was to identify the critical nutrimental parameters among a large number of variables with a significant effect either positively or negatively on ALP production through shake-flask submerged cultivation of *Lysinibacillus* sp. strain APSO concerning their main effect. Unless such a predictive model approach has been used, no interaction between various influences in the range of variables under consideration is assumed. By this, nine independent variables [molasses, NaNO_3_, (NH_4_)_3_SO_4_, eggshell, NaCl, MgCl_2_·6H_2_O, CuSO_4_·5H_2_O, CoCl_2_·6H_2_O, and ZnSO_4_·H_2_O] were screened with three center points in 23 combination trials with the corresponding response (ALP activity) to generate regression coefficient values. For each, the independent variable was evaluated at three levels. All experiments were duplicated to estimate the standard deviation, and the means of ALP productivity were taken as a response. Table 1 displays the list of variables under study and their coded and actual levels as well as the layout of the design matrix, illustrating that each variable is equal at high and low levels 10 times in each column. The following first-order polynomial approach was used for mathematical modeling for the screening process:

1 $${\rm{Y}}_{{{\rm{ALP}}\;{\rm{activity}}}} = \, \beta_{{\rm{o}}} + \sum \beta_{{\rm{i}}} {\rm{X}}_{{\rm{i}}}$$

where Y is the predicted response (ALP activity; U L^−1^ min^−1^), β_o_ is the model intercept, β_i_ is the linear regression coefficient, and X_i_ is the coded independent variable estimate. The design allows the estimation of the main influence of the variables analyzed and manages data in a rank order depending on the magnitude and sign of the effects.

The experimental design and statistical analysis of the data were done using the essential experimental design free software to estimate *t*-values, *ρ*-values, and confidence levels as a percent expression of the *ρ*-value via ANOVA. The Student’s *t*-test was used to evaluate the significance level (*p*-value) of each concentration effect:

2 $${\rm{t}}_{{({\rm{xi)}}}} = {\rm{E}}\left( {{\rm{X}}_{{\rm{i}}} } \right)/{\rm{SE}}$$

The standard error (SE) is the square root of the variance of the factor effect. Factors with the highest *t*-values and confidence levels above 95% (*p* < 0.05) were considered extremely significant on ALP productivity. To check the significance and the fitness of the obtained regression model, the *R* and *R*^2^ coefficients were calculated. The Pareto diagram was utilized for ranking the independent variables according to their significant effects on productivity. From the Pareto chart, factors that exhibited the highest positive effects (X_1_, molasses; X_2_, NaNO_3_; and X_6_, MgCl_2_·6H_2_O) were picked for further optimization using CCD-uniform precision^36^. A validation test was carried out in which the predicted optimal levels of the independent variables were examined and compared to the basal condition setting, and the average enzyme production was calculated.

### RSM (CCD-uniform precision)

To elucidate the cumulative interactive effects of the most significant independent macronutrients of the medium and optimize their concentration to enhance ALP production, RSM was used in combination with CCD. RSM combined computational and mathematical technologies to make modeling and analysis accessible^37,38^. Based on the findings from PBD, CCD-uniform precision of 2^3^ = eight cube points plus six center points and six axial points was utilized to further improve the three variables that exhibited significant positive effects on ALP productivity by *Lysinibacillus* sp. strain APSO and had the highest percentage of contribution. In Table 2, the independent variables of the highest confidence levels, namely, molasses (X_1_), NaNO_3_ (X_2_), and MgCl_2_·6H_2_O (X_3_), were studied at five experimental levels (− α, − 1, 0, + 1, and + α), where α = 16,817. Based on the principle of CCD design, combinations of the three independent significant variables were executed in 20 experiments, as shown in Table 3. The following equation explains the interrelationship between coded values and actual values:

3 $${\rm{X}}_{{\rm{i}}} = {\rm{X}}_{{\rm{i}}} - {\rm{X}}_{{{\rm{cp}}}} /\Delta {\rm{x}}_{{\rm{i}}}$$

where X_i_ is the dimensionless value of independent variables, x_i_ is the real value of independent variables, x_cp_ is the real value of independent variables at the center point, and Δx_i_ is the step change of the real value of variable i representing a variation of a unit for dimensionless value of variable i. The interrelationship between independent variables, their correlations, and their responses was calculated in the quadratic equation using the second-order polynomial model:

4 $${\rm{Y}} = \beta_{0} + \, \Sigma_{{\rm{i}}} \beta_{{\rm{i}}} {\rm{X}}_{{\rm{i}}} + \, \Sigma_{{{\rm{ij}}}} \beta_{{{\rm{ij}}}} {\rm{X}}_{{\rm{i}}} {\rm{X}}_{{\rm{j}}} + \, \Sigma_{{{\rm{ii}}}} \beta_{{{\rm{ii}}}} {\rm{X}}_{i}^{2}$$

where Y is the predicted response (ALP activity; U L^−1^ min^−1^), β_0_ is the model intercept, X_i_, and X_j_ are the independent variables, β_i_ is the linear coefficient, β_ij_ is the cross-product coefficient, and β_ii_ is the quadratic coefficient. Laboratory verification was conducted to develop an equation model and ensure an accurate calculation of the theoretical values of each variable.

All data generated from the factorial experiment were analyzed using least squares to construct the regression models. Experimental design, data analysis, interaction plotting, and optimization of factor conditions were achieved using essential experimental design free software. The solver function of the Microsoft Excel tool was utilized to validate model fits throughout the case of predicted responses to experimentally derived data. For the estimation of interaction between different variables, the 3D response surface plots were built via Statistica 7.0 software, and the point prediction method was used to identify the appropriate levels for each variable. The response surface chart with the vertical axis reflecting enzyme activity and two horizontal axes representing five different levels of both explicative nutrients were generated for the visual evaluation of the overall response pattern and the interactive influence of the significant variables on the response, whereas one other factor was held at zero levels. ANOVA was exploited to ensure a quality model through a series of essential measurements, such as the sum of squares (SS) used to estimate the factor’s main effects, *F*-ratios as the ratio of the respective mean square effect, and mean square error (MS).

### Scale-up production of bacterial ALP

The scale-up of a bioprocess is a critical stage that guarantees the economic viability of the bioproduct concerned and is considered an important link for the bioprocess transfer from the laboratory scale to the industrial scale for commercial needs^39^. The primary goal of this study was to establish a large-scale fermentation system to evaluate microbial growth kinetics in a submerged cultivation system.

### Shake-flask cultivation system

Batch-mode shake-flask experiments were implemented in 250 mL Erlenmeyer flasks containing 50 mL optimized medium (molasses, 28.233 g L^−1^; NaNO_3_, 0.645 g L^−1^; MgCl_2_·6H_2_O, 0.417 g L^−1^; pH 7.0 ± 2) at 45 °C in a shaker incubator (200 rpm) for 30 h. The production medium was inoculated with 1% (v/v) of 12 h old activated preculture suspension. The samples were taken out periodically every 2 h, and cell growth was monitored by measuring the absorbance at 600 nm against a blank (optimized medium without inoculation) using a Beckman DU spectrophotometer. The bacterial culture was centrifuged at 10,000 rpm for 10 min under cooling conditions to obtain cell-free supernatants. Then, the following analytical parameters were monitored throughout the incubation period: biomass dry weight, ALP activity, total soluble phosphate concentration, total carbohydrate concentration, and total soluble protein concentration. All experiments were executed in triplicate.

### Stirred-tank bioreactor batch cultivation system

To validate the optimized fermentation medium for ALP production at a large scale, bioreactor cultivations were executed in a 7.0 L (4.0 L working volume) stirred-tank bioreactor (Bioflo 310) in which 3960 mL statistically optimized medium was sterilized by autoclaving at 121 °C for 15 min. The agitator was equipped with two six-bladed Rushton turbine impellers and four baffles. The agitation speed was 200 rpm throughout cultivation. Aeration was performed using filtered sterilized compressed air, which was supplied continuously to the bioreactor at a rate of 0.5 VVM. The scale-up process was automated with a Bio-command multiprocess control software supported by the control panel of a 10.4 color touch-screen interface computer system. The system unit of the bioreactor was equipped with a digitally controlled pH electrode, temperature probe, and polarographic DO electrode (Ingold, Mettler-Toledo, Switzerland). The set points for temperature and pH value were 45 °C and 7.0, respectively. Then, 1% of the log-phase activated precultured inoculum was added aseptically to the bioreactor vessel with the aid of the inoculation bottle. The silicon-based antifoam grade A (Sigma-Aldrich, Inc., USA) was added manually to hold back the foam formation when necessary. In the pH-controlled batch cultivation system, pH was controlled by automatic feeding of 2 N NaOH and 2 N HCl via a peristaltic pump. Throughout the fermentation duration, a 20 mL actively growing culture was drawn out periodically at regular intervals in 50 mL sterile Falcon tubes. The optical density measurement for growing cell cultures was achieved at 600 nm using a UV–vis spectrophotometer. Centrifugation of the withdrawn culture samples was performed at 10,000 rpm for 10 min under cooling conditions to obtain cell-free supernatants that were then exploited for further analytical procedures.

### Analytical procedures

#### Determination of biomass dry weight

A certain volume of the fermentation broth (10 mL) was withdrawn at different intervals during the fermentation process in preweighed 15 mL sterile Falcon tubes and centrifuged at 10,000 rpm for 10 min. The pellets containing cell debris were washed with physiological saline twice, centrifuged, and dried to constant weight at 80 °C. The correlation between biomass dry weight and optical density (OD_600_) of the bacterial culture was constructed to estimate the correlation factor (δ).

### Estimation of total carbohydrate concentration

The total carbohydrate concentration in the culture filtrate was estimated quantitatively by the anthrone sulfuric acid method of Morris et al.^40^. The standard curve was established by 0.1 mg mL^−1^ sucrose solution stock. The principle of the reaction is to hydrolyze the polysaccharides and hydrate the monosaccharides to form furfural from pentoses and hydroxymethylfurfural from hexoses as a result of the action of sulfuric acid under boiling conditions. The green color was the outcome of the reaction of the solutions of furfural and hydroxylfurfural with an anthrone reagent, which was measured spectrophotometrically at 620 nm.

### Determination of total soluble phosphate concentration

The quantitative estimation of the dissolved phosphate concentration in the culture filtrate was done using an ascorbic acid-molybdenum blue method of Chen et al.^41^. The development of the molybdenum blue color intensity was measured at 820 nm, and 100 µg mL^−1^ potassium dihydrogen phosphate solution stock was used for building up the standard curve.

### Protein concentration assay

Lowry procedure was used to estimate the total soluble protein content in the culture filtrate using bovine serum albumin (Sigma) for the standard curve construction^42^.

### Atomic absorption analysis

Cell-free supernatants were subjected to AAS (Zeenit 700, Analytik Jena, Germany) at the Laboratory Centre SRTA-City to determine the residual concentrations of heavy metals in the culture filtrate using a standard method^43^.

## Supplementary Information

Supplementary Information.

## Data Availability

All data produced during this study are included in this published article.
