# Supplementary figures and images for: Biovalorization of raw agro-industrial waste through a bioprocess development platform for boosting alkaline phosphatase production by Lysinibacillus sp. strain APSO
Source: Sci Rep. 2021 Sep 2;11:17564. doi: 10.1038/s41598-021-96563-6 (PMC8413444; doi:10.1038/s41598-021-96563-6)

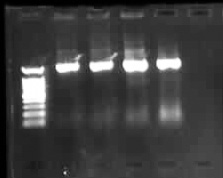


**1500 bp**

S.1. Agarose gel electrophoresis photogram for the purified PCR product of 16S rRNA gene.

Supplement: Supplementary file 1 — Supplementary Information. [file 41598_2021_96563_MOESM1_ESM.docx]
